# Robust FDTD Modeling of Graphene-Based Conductive Materials with Transient Features for Advanced Antenna Applications

**DOI:** 10.3390/nano13030384

**Published:** 2023-01-18

**Authors:** Pablo H. Zapata Cano, Stamatios Amanatiadis, Zaharias D. Zaharis, Traianos V. Yioultsis, Pavlos I. Lazaridis, Nikolaos V. Kantartzis

**Affiliations:** 1 School of Electrical and Computer Engineering, Aristotle University of Thessaloniki, 54124 Thessaloniki, Greece; 2 School of Computing and Engineering, University of Huddersfield, Huddersfield HD1 3DH, UK

**Keywords:** FDTD methods, gas sensing, graphene, graphene oxide antenna, transient phenomena

## Abstract

The accurate modeling of frequency-dispersive materials is a challenging task, especially when a scheme with a transient nature is utilized, as it is the case of the finite-difference time-domain method. In this work, a novel implementation for the modeling of graphene-oriented dispersive materials via the piecewise linear recursive convolution scheme, is introduced, while the time-varying conductivity feature is, additionally, launched. The proposed algorithm is employed to design a reduced graphene-oxide antenna operating at 6 GHz. The transient response to graphene’s conductivity variations is thoroughly studied and a strategy to enhance the antenna performance by exploiting the time-varying graphene oxide is proposed. Finally, the use of the featured antenna for modern sensing applications is demonstrated through the real-time monitoring of voltage variation.

## 1. Introduction

Since the discovery of isolated and stable graphene films [1], graphene has gained the interest of the entire research community, due to its promising mechanical, thermal, and optical properties, which include sufficient electrical conductivity, high optical transmittance, elevated Young’s modulus, and thermal conductivity, among others [2,3,4,5,6]. During the last few years, graphene has been applied to the design of various contemporary devices, including FETs, diodes, absorbers, and attenuators or antennas [7,8,9,10,11,12]. In essence, many of these components exploit the electrically tunable conductivity of graphene, as well as its variable Fermi level or the concatenation of its plasmonic resonances to enable some functionalities, such as radio-frequency identification (RFID), terahertz and microwave absorption [13,14], radio fre beam steering, or interference mitigation [15]. Among the existing methods to obtain graphene samples, the simple top-down synthesis of chemically modified graphene materials, such as the graphene oxide (GO) or the reduced graphene oxide (rGO), provides instructive opportunities for an easier and more feasible fabrication [6]. The first efforts to oxidize graphite were carried out back in 1859 along with the respective explorations of the graphite structure [16]. Since this significant discovery, many oxidation techniques have been proposed, the most popular of which is the Hummers’ method [17] and its subsequent modified versions. Basically, they are based on the reaction of graphite with a protonated solvent (concentrated sulfuric acid, H${}_{2}$SO${}_{4}$) and a strong oxidizing agent (usually KMnO${}_{4}$) [18]. The presence of functional groups containing oxygen leads to the presence of π-electrons that decrease the carrier mobility and carrier concentration. This makes GO insulating and thermally unstable by nature [19]. In order to tackle this hindrance and create materials with properties similar to those of pristine graphene, several efforts have been, recently, made towards the removal of the GO oxygen functional groups via a process designated as “reduction”, which enables the production of reduced rGO. This procedure can be implemented in terms of diverse means which result in different rGO properties with a critical impact on the performance of the relative [20]. The promising properties and synthesis simplicity of the GO and rGO has paved the way to many industrial applications, whose electrical, thermal, and mechanical attributes can be profitably exploited [21], such as membranes and coatings, thermal/light/humidity actuators, anti-corrosive coatings, and batteries or sensors. Moreover, its capacity to remove water pollutants makes GO an attractive candidate to build adsorbent for environmental cleanup [22].

In particular, the research for efficient graphene-oxide-based sensors has, lately, escalated, owing to their remarkable properties [23,24,25]. Actually, the elevated surface-to-volume ratio allows for the detection of a species with a sensitivity level down to a single atom or molecule (quantum detection). This feature of the graphene oxide together with its extremely high electrical conductivity (even in the presence of few carriers) and low noise permit a detectable change in resistance even down to the sub-ppm and ppb level of the target species [26]. It should be stressed that the conductivity of graphene varies upon exposure to the target species (gas/vapor). These sensing species act as temporary dopants to the graphene layer and alter its localized electronic concentration, contributing, thereby, either electrons (such as in NH${}_{3}$ or CO) or holes (like in H${}_{2}$O or NO${}_{2}$). On the other hand, metal oxide semiconductors (MoSs) have been widely used for gas detection [27,28], although they have very large recovery times (or even no recovery at all). Moreover, hybrid GO and MoS structures [29,30] are proven to be viable alternatives to MoS-based sensors, while their rGO-based counterparts can effectively detect chemically aggressive gases (i.e., NO${}_{2}$, NH${}_{3}$, Cl${}_{2}$, and NO) with typical response and recovery times of about several tens of minutes [31,32]. Finally, the electronic and mechanical properties of graphene enable an easier transduction of the sensing signal, turning the rGO to a very attractive candidate for the design of efficient sensing devices [33,34,35].

Essentially, the aforementioned applications rely on the appropriate modeling of the involved media. In general, the characterization of graphene-based materials is achieved through their surface conductivity. Specifically, graphene itself is considered as a truly two-dimensional layer and its conductivity is evaluated via the Kubo formula [36]. The latter is described by means of a Debye function until the far-infrared regime, while beyond that limit a more complicated pattern is used in terms of Lorentz series. Concerning the rGO, it is, also, considered as a planar material up to the THz spectrum, since its electrical thickness is negligible at these frequencies. Consequently, it is defined by its surface conductivity that is almost constant at this regime approximating the Debye model [21,37].

Nevertheless, the analysis of modern devices is not feasible through analytical solutions; therefore, the surface conductivity features must be incorporated into powerful numerical algorithms, such as the finite-difference time-domain (FDTD) method [38]. The latter is particularly popular due to its transient nature that enables both the broadband analysis and inclusion of time-varying attributes. However, the combination of planar material modeling with frequency dispersion is not a trivial procedure. Various works have been conducted focusing on graphene, where its 2D nature is implemented as a surface impedance [39], an an equivalent surface current controlled explicitly by the electric field [40] or inserting its contribution through the magnetic field boundary conditions [41]. Moreover, these approaches resolve the frequency dispersion using the auxiliary differential equation (ADE), whereas the recursive convolution method (RCM) proved advantageous for specific applications [42]. Hence, it would be very interesting and instructive to examine the piecewise linear recursive convolution (PLRC) technique [43] that retains the benefits of the conventional RCM, yet exhibits a second-order accuracy like the ADE.

Based on these aspects, the main contribution of this work is the development of a novel scheme for the modeling of graphene-oriented conductive materials with frequency-dispersive response in the popular FDTD method. The transient nature of the latter is exploited to enable the simulation of planar materials with time-varying features and properties, which offers many possibilities for the design of complex devices for advanced applications. Moreover, the frequency dispersion is handled using an efficient PLRC algorithm that is appropriately adjusted to the surface conductivity function of the involved materials. Then, the developed implementation is applied to the design of an rGO antenna. An observation of the rGO transient response for sensing applications is performed, and some concepts for the enhancement of the device’s response are proposed by means of the time-varying rGO traits.

The rest of the paper is organized as: Initially, the featured FDTD scheme for the modeling of the rGO is introduced in Section 2, together with a validation of the implementation. The second part of the work is devoted to the applications of the proposed model. Thus, in Section 3 an rGO antenna is designed, and a thorough study on the impact of graphene’s conductivity variations on the antenna performance and sensing capabilities is conducted.

## 2. FDTD Modeling of Time-Varying Planar Conductive Materials

**Figure 1:**
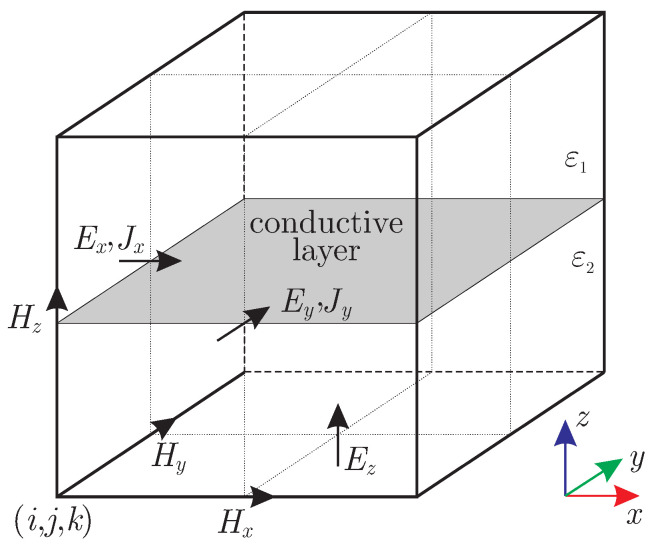
Conductive layer modeling in the Yee cell as an equivalent surface current density.

Two challenging tasks are encountered for the effective transient modeling of frequency-dependent conductive layers, such as the graphene, the GO, and the rGO. Initially, we consider that any planar material is characterized through its surface conductivity $\sigma_{p}\left(\omega \right)$ and it is infinitesimally thin. The latter is rational since the thickness of such materials is several orders of magnitude smaller compared to the wavelength, even at the infrared regime. To this end, the representation in the FDTD algorithm is realized via the equivalent surface $J_{p}$:

(1) $$J_{p}\left(\omega \right)=\sigma_{p}\left(\omega \right)E\left(\omega \right),$$

where E is the electric field at the same region. For this reason, the conductive layer is placed as depicted in Figure 1, considering its orientation at the xy-plane, while the surface current components are allocated at the identical point to the electric ones. Now, the integral form of the Ampère’s law is reviewed at a normal, to the conductive sheet, plane, e.g., the xz one:

(2) $$\oint_{C}H\cdot dl=\underset{S}{\;\int \int \;}\varepsilon \frac{\partial E}{\partial t}\cdot dS+\underset{S}{\;\int \int \;}J_{p}\delta \left(z\right)\cdot dS.$$

Here, we included the Dirac delta function, δ(z), to preserve the planar nature of the surface current. Upon discretizing Equation (2), using the finite-difference scheme, we conclude to the electric field update equation that incorporates the planar material contribution:

(3) $$\begin{array}{cc} E_{y}{|}_{i+\frac{1}{2},j,k+\frac{1}{2}}^{n+1}= & E_{y}{|}_{i+\frac{1}{2},j,k+\frac{1}{2}}^{n}+\frac{2\Delta t}{\varepsilon_{1}+\varepsilon_{2}}\left(\frac{H_{x}{{|}_{i+\frac{1}{2},j,k+1}^{n+\frac{1}{2}}-H_{x}|}_{i+\frac{1}{2},j,k}^{n+\frac{1}{2}}}{\Delta z}+\right.\\ & +\left.\frac{H_{z}{{|}_{i,j,k+\frac{1}{2}}^{n+\frac{1}{2}}-H_{z}|}_{i+1,j,k+\frac{1}{2}}^{n+\frac{1}{2}}}{\Delta x}\right)+\frac{2\Delta t}{(\varepsilon_{1}+\varepsilon_{2})\Delta z}J_{p,y}{|}_{i+\frac{1}{2},j,k+\frac{1}{2}}^{n}. \end{array}$$

It is important to mention that the δ(z) function of the electrically-controlled current is converted to Δz. Therefore, the current density $J_{p}$ is normalized to the desired equivalent surface current term of our conductive sheet. Observe that this modification is applied to the electric components that include the planar material, while all the remaining ones are evaluated via the original FDTD scheme, retaining, hence, the overall algorithmic efficiency.

Now, the second challenge appears, namely the robust implementation of the frequency dispersion for the conductive layer. In particular, the second-order accurate PLRC method is modified appropriately to model the surface current dispersion. First of all, the transition of Equation (1) into the time-domain is obtained as:

(4) $$J_{p}\left(t\right)=\sigma_{p}\left(t\right)*E\left(t\right),$$

where ∗ corresponds to the convolution. In the continuous space, Equation (4) is expanded into:

(5) $$J_{p}\left(t\right)=\int_{\zeta =0}^{t}\sigma_{p}\left(\zeta \right)E\left(t-\zeta \right)d\zeta ,$$

with ζ connected to the integration depth towards the previous electric field values. Then, the discrete form is calculated using the time-step Δt:

(6) $$J_{p}{|}^{n}=\int_{\zeta =0}^{n\Delta t}\sigma_{p}\left(\zeta \right)E\left(n\Delta t-\zeta \right)d\zeta .$$

Note that the continuous variation inside the integral term is maintained through the assumption that the electric field is linearly progressing via the different time-steps:

(7) $${E\left(t\right)=E|}^{n}+\frac{{E|}^{n+1}{-E|}^{n}}{\Delta t}\left(t-n\Delta t\right),\;n\Delta t⩽t⩽\left(n+1\right)\Delta t.$$

Therefore, the continuous integration can be derived at each time-step where the electric field values are constant, i.e.,

$$J_{p}{|}^{n}=\sum_{i=0}^{n-1}\int_{\zeta =i\Delta t}^{(i+1)\Delta t}\sigma_{p}\left(\zeta \right)\left[{E|}^{n-i}+\frac{{E|}^{n-i-1}{-E|}^{n-i}}{\Delta t}\left(\zeta -i\Delta t\right)\right]d\zeta ,$$

(8) $$J_{p}{|}^{n}=\sum_{i=0}^{n-1}\left[{E|}^{n-i}\left(\int_{\zeta =i\Delta t}^{(i+1)\Delta t}\sigma_{p}\left(\zeta \right)d\zeta \right)+\frac{{E|}^{n-i-1}{-E|}^{n-i}}{\Delta t}\left(\int_{\zeta =i\Delta t}^{(i+1)\Delta t}\sigma_{p}\left(\zeta \right)\left(\zeta -i\Delta t\right)d\zeta \right)\right].$$

By setting the continuous integration terms as:

(9) $$\chi^{i}=\int_{\zeta =i\Delta t}^{(i+1)\Delta t}\sigma_{p}\left(\zeta \right)d\zeta ,\;\;\xi^{i}=\int_{\zeta =i\Delta t}^{(i+1)\Delta t}\sigma_{p}\left(\zeta \right)\left(\zeta -i\Delta t\right)d\zeta ,$$

the surface current update equation Equation (8) is simplified to:

(10) $$J_{p}{|}^{n}=\sum_{i=0}^{n-1}\left[{E|}^{n-i}\chi^{i}+{\left(E\right|}^{n-i-1}-E{|}^{n-i})\xi^{i}\right].$$

This formula indicates that all the previously stored electric field values are required for the calculation of Equation (10), degrading significantly the FDTD performance. Nevertheless, a recursive computation scheme is applied for the case of time-domain conductivity functions that can be represented via series of exponential terms. Interestingly, various popular dispersion models, such as the Debye, Lorentz and Drude functions, are compatible to the aforementioned condition. So, Equation (10) is calculated for this class of functions via:

$$J_{p}{|}^{n}{=E|}^{n}\chi^{0}+{\left(E\right|}^{n-1}-E{|}^{n})\xi^{0}+\sum_{i=1}^{n-1}\left[{E|}^{n-i}\chi^{i}+{\left(E\right|}^{n-i-1}-E{|}^{n-i})\xi^{i}\right],$$

$$J_{p}{|}^{n}{=E|}^{n}\chi^{0}+{\left(E\right|}^{n-1}-E{|}^{n})\xi^{0}+\sum_{i=0}^{n-2}\left[{E|}^{n-i-1}\chi^{i+1}+{\left(E\right|}^{n-i-2}-E{|}^{n-i-1})\xi^{i+1}\right],$$

(11) $$J_{p}{|}^{n}{=E|}^{n}\chi^{0}+{\left(E\right|}^{n-1}{-E|}^{n}{)\xi^{0}+C_{\mathrm{rec}}J_{p}|}^{n-1},$$

where $C_{\mathrm{rec}}=\chi^{i+1}/\chi^{i}=\xi^{i+1}/\xi^{i}$ is a recursion constant and

(12) $$J_{p}{|}^{n-1}=\sum_{i=0}^{n-2}\left[{E|}^{n-i-1}\chi^{i}+{\left(E\right|}^{n-i-2}-E{|}^{n-i-1})\xi^{i}\right].$$

Next, the proposed methodology is applied to the modelling of an rGO layer. Its conductivity is evaluated by the Debye function of [37]

(13) $$\sigma_{\mathrm{rGO}}\left(\omega \right)=\frac{\varepsilon_{0}\omega_{p}^{2}\tau }{1+j\omega \tau },$$

with $\varepsilon_{0}$ the free-space dielectric permittivity, $\omega_{p}$ the plasma frequency, and τ the relaxation time. The transition into the time-domain results in the exponential function:

(14) $$\sigma_{\mathrm{rGO}}\left(t\right)=\varepsilon_{0}\omega_{p}^{2}e^{-\frac{t}{\tau }}u\left(t\right),$$

where u(t) is the unit step-function. The continuous integration terms $\chi^{i}$ and $\xi^{i}$ are calculated from Equation (9):

(15) $$\chi^{i}=\varepsilon_{0}\omega_{p}^{2}\tau \left(1-e^{-\frac{\Delta t}{\tau }}\right)e^{-\frac{i\Delta t}{\tau }},\;\xi^{i}=\frac{\varepsilon_{0}{\left(\omega_{p}\tau \right)}^{2}}{\Delta t}\left[1-\left(\frac{\Delta t}{\tau }+1\right)e^{-\frac{\Delta t}{\tau }}\right]e^{-\frac{i\Delta t}{\tau }}.$$

It is obvious that the exponential term leads to the convenient computation of the recursion constant:

(16) $$C_{\mathrm{rec}}=\frac{\chi^{i+1}}{\chi^{i}}=\frac{\xi^{i+1}}{\xi^{i}}=e^{-\frac{\Delta t}{\tau }},$$

while the required $\chi^{0}$ and $\xi^{0}$ are derived:

(17) $$\chi^{0}=\varepsilon_{0}\omega_{p}^{2}\tau \left(1-C_{\mathrm{rec}}\right),\;\xi^{0}=\frac{\varepsilon_{0}{\left(\omega_{p}\tau \right)}^{2}}{\Delta t}\left[1-\left(\frac{\Delta t}{\tau }+1\right)C_{\mathrm{rec}}\right].$$

Finally, the time-domain nature of the FDTD method enables the powerful feature of transient conductivity alteration, a feature that is absent from the majority of the commercial packages. Particularly, the conductivity of various planar materials can be adjusted externally, such as the electrostatic bias of graphene or the gas and humidity dependence of rGO [33]. As a consequence, this real-time material parameter alteration leads to the investigation of several interesting phenomena, including the non-linear device response. As an example, the surface conductivity $\sigma_{\mathrm{rGO}}$ of rGO can be periodically modulated by means of:

(18) $$\sigma_{\mathrm{rGO}}\left(t\right)=\varepsilon_{0}\omega_{p}^{2}e^{-\frac{t}{\tau }}u\left(t\right)\left[1+m_{0}\sin\left(\omega_{m}t\right)\right],$$

where $m_{0}$ and $\omega_{m}$ are the modulation strength and frequency, respectively. Then, the transition to the discrete space requires the updating of $\chi^{0}$ and $\xi^{0}$ terms at each time-step:

(19a) $$\chi^{0}=\varepsilon_{0}\omega_{p}^{2}\tau \left(1-C_{\mathrm{rec}}\right)\left[1+m_{0}\sin\left(\omega_{m}n\Delta t\right)\right],$$

(19b) $$\xi^{0}=\frac{\varepsilon_{0}{\left(\omega_{p}\tau \right)}^{2}}{\Delta t}\left[1-\left(\frac{\Delta t}{\tau }+1\right)C_{\mathrm{rec}}\right]\left[1+m_{0}\sin\left(\omega_{m}n\Delta t\right)\right].$$

**Figure 2:**
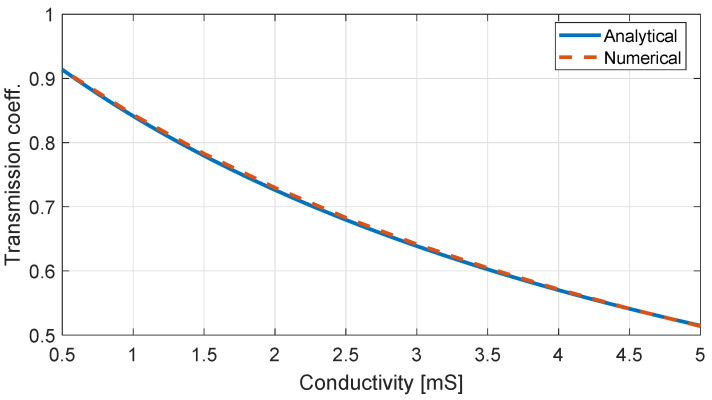
Comparison between the theoretical to the numerically extracted transmission coefficient for the propagation towards an infinite rGO layer varying its conductivity.

The previously described scheme is verified in terms of its accuracy via its thorough comparison with analytical solutions as well as with well-established a numerical software. Firstly, a finite rGO layer is considered and the transmission coefficient is evaluated at microwave frequencies. The conductivity of the rGO at this regime is almost constant; thus, the relaxation time is selected to be τ=4.8 ps while the plasma frequency is selected properly to approximate a surface conductivity at the range of 0.5–5 mS. Then, the analytical value for the transmission coefficient is calculated through

(20) $$\left|T\right|=\frac{2}{2+\sigma_{\mathrm{rGO}}\eta_{0}}$$

where $\eta_{0}$ is the free-space wave impedance. The comparison to the numerically extracted results is depicted in Figure 2, proving a remarkable matching.

**Figure 3:**
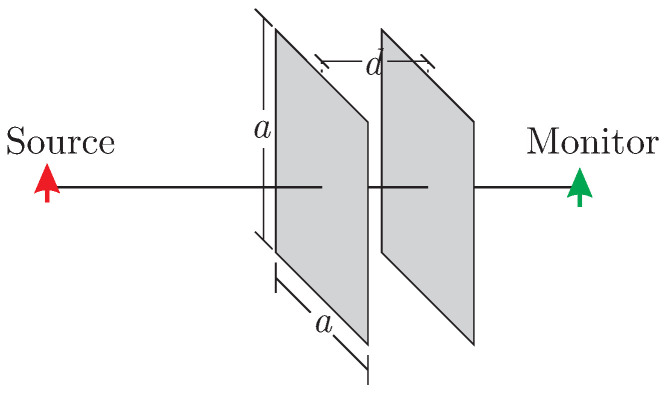
Setup of two finite rGO layers at distance *d*.

**Figure 4:**
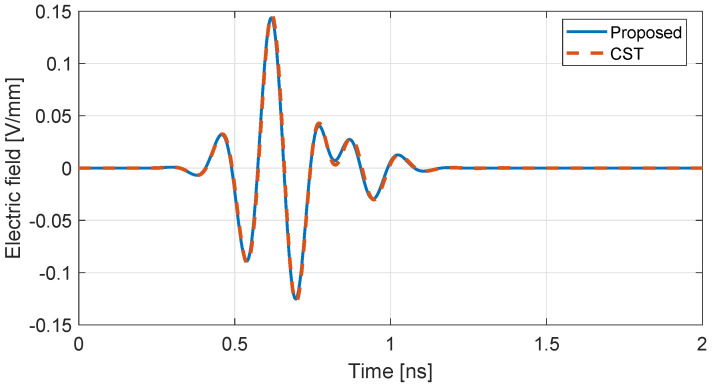
Comparison of the electric field for the setup in Figure 3 between the proposed scheme and the CST commercial software.

Additionally, the more complex setup of Figure 3 is examined, where two 50×50 mm${}^{2}$ rGO sheets are placed at the distance of d=7.6 mm. The source is tangential to the layers, while an electric field monitor with identical orientation is placed behind the second layer. The numerical results of the proposed algorithm are compared to those provided by the well-established CST Studio Suite [44], where the planar conductive material is treated as a surface impedance. The results, given in Figure 4 highlight the influence of multiple reflections between the rGO layers after 0.8 ns, Moreover, the two curves are practically in full agreement, verifying successfully the correct implementation of surface conductive materials through the proposed PRLC method.

Finally, the featured modeling methodology is compared to some well-established techniques in Table 1 that are able to handle frequency-dependent materials via diverse schemes. In particular, the ADE and PLRC ones achieve a second-order accuracy, while their approximation is equivalent to those attained by techniques that treat the planar conductivity as a surface impedance. Note that the original PLRC scheme in [43] does not provide the extension to planar materials, which is implemented in the current work. Moreover, the time-varying properties of a dispersive material are effectively enabled in our algorithm, thus offering a novel and instructive numerical feature.

## 3. Design of an rGO-Enhanced Patch Antenna

In this section, the newly introduced modeling of planar conductive materials is applied on the design of a patch antenna that includes an rGO patch. The transient nature of the method is exploited to perform an analysis on the effect of the planar material’s conductivity changes on the performance of the antenna. Moreover, it is shown how the conductivity variations can result on an enhancement of the antenna radiation characteristics, and, thus, can be used for sensing purposes.

### 3.1. Antenna Parameters and Simulation Properties

**Figure 5:**
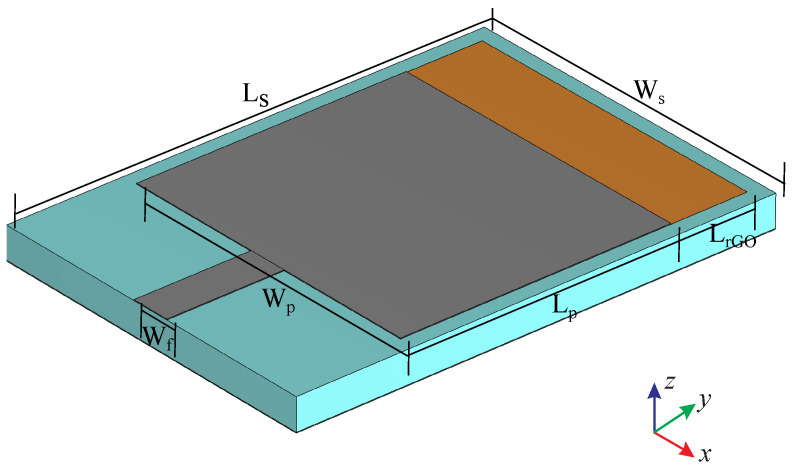
Geometry of the proposed rGO antenna, with $W_{f}=2.5$ mm, $W_{p}=15,$ mm, $W_{s}=19.5$ mm, $L_{p}=17.5$ mm, $L_{\mathrm{rGO}}=5$ mm, and $L_{s}=28.5$ mm.

The proposed patch antenna has the dimensions depicted in Figure 5 and is designed to operate in the vicinity of 6 GHz. Both the metal patch and the rGO are deposited on a substrate made of a 2 mm-thick Teflon material with an electric permittivity of ε=2.33. Moreover, the computation domain is divided into 56×56×56 cells of Δx=Δy=2.5 mm and Δz=2 mm, with a time-step of Δt=4.4 ps, while the open boundaries are truncated by an 8-cell thick perfectly matched layer (PML) [38,42,45].

### 3.2. Antenna Response through the Variation of the rGO Conductivity

**Figure 6:**
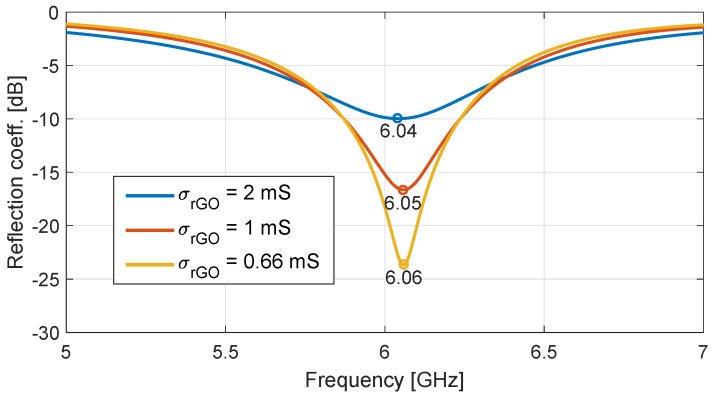
Response of the proposed antenna for different constant rGO conductivity values.

Firstly, we investigate the effect of rGO conductivity changes on the antenna response, both for constant and time-modulated values of $\sigma_{rGO}$. Thus, Figure 6 depicts the impact of the $\sigma_{rGO}$ variation on the antenna reflection coefficient. As observed, both a quality factor variation and a frequency shift are produced at the resonant pick when the conductivity is modified. More precisely, a 10 MHz shift occurs when $\sigma_{rGO}$ varies from 2 to 1 mS.

**Figure 7:**
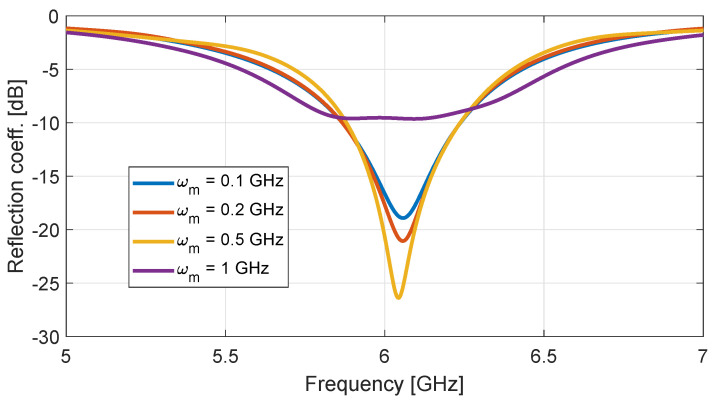
Response of the proposed antenna with a time-modulated conductivity, as in Equation (18), for different modulation frequencies $\omega_{m}$ and $m_{0}=0.5$, $\varepsilon_{0}\omega_{p}^{2}=1$ mS.

Subsequently, taking avantage of the transient nature of the FDTD method, $\sigma_{rGO}$ is time-modulated according to Equation (18). Both the modulation strength, $m_{0}$, and the central value of the conductivity are fixed to 0.5 and 1 mS, respectively. Then, the modulation frequency, $\omega_{m}$, can vary from 0.1 to 1 GHz and the resulting antenna reflection coefficient is shown in Figure 7.

**Figure 8:**
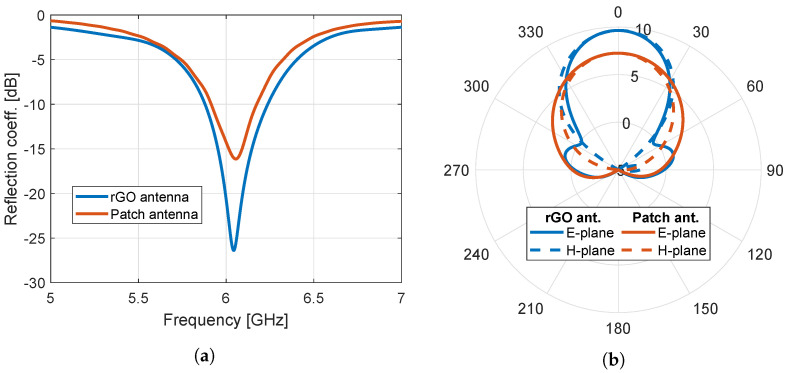
Performance assessment of the proposed time-modulated rGO antenna. (**a**) Response of the developed structure ($\omega_{m}$ = 0.5 GHz) and its equivalent patch antenna and (**b**) radiation pattern of both antennas at the resonant frequency of 6.04 GHz. The directivity values are in dBi.

On the other hand, the variation of the modulation frequency leads to different antenna responses. Explicitly, the deepest resonant pick is reached for a modulation frequency of $\omega_{m}=$ GHz, attaining a reflection coefficient of −26.37 dB at the resonance. Actually, this constitutes a very significant improvement of the antenna radiating performance, showcased in Figure 8, where the directivity is, also, enhanced by approximately 2 dBi.

**Figure 9:**
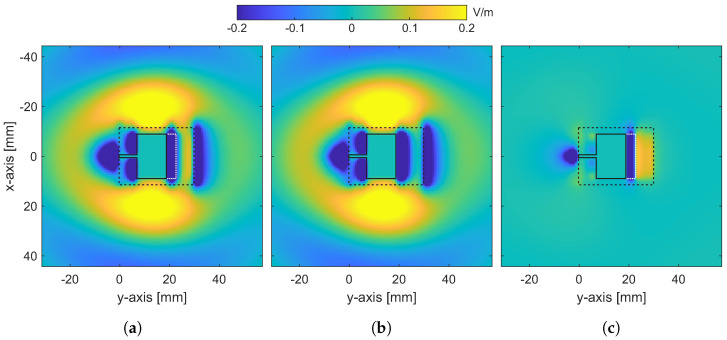
Distribution of the $E_{y}$ electric field component for (**a**) the proposed rGO, (**b**) the conventional metallic patch antenna and (**c**) their subtraction [dashed black line: outline of the substrate layout; solid black line: outline of the metal patch; white dotted line: outline of the rGO layer].

It becomes apparent that the optimal rGO antenna with the time-modulated conductivity exhibits a definite improvement of around 10 dB in the resonance depth with respect to the conventional patch antenna. Additionally, the electric field distribution for both antennas is respectively extracted in Figure 9a,b for the $E_{y}$ component on the xy-plane that matches to the upper side of the substrate. To probe further, Figure 9c illustrates the subtraction of the aforementioned distributions. As observed, the presence of the rGO layer in front of the metallic patch decreases the field concentration on its surface, while augmenting it towards the +y-axis. All these aspects can be considered to explain the optimal behaviour of the proposed device in terms of the resonance depth and the enhanced directivity.

Moreover, the total efficiency (gain) is calculated as 98.7% (9.58 dBi) and 96.4% (7.23 dBi) for the time-modulated rGO antenna and the plain metal patch, respectively, at their resonant frequency proving, again, the advantageous performance of the proposed device. As a result, some interesting possibilities are unveiled for the enhancement of the antenna response, in which time-modulated conductivities can be practically realized, for instance, via the induction of a time-changing bias voltage on the rGO plate, which has a direct impact on the conductivity.

### 3.3. Transient Response for Sensing Applications

**Figure 10:**
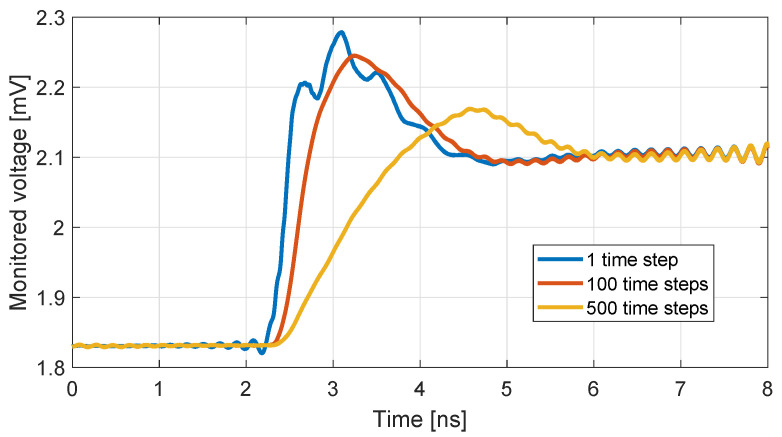
Time evolution of the monitored voltage when $\sigma_{\mathrm{rGO}}$ varies from 2 to 1 mS.

As already demonstrated in Figure 6, a shift of the resonant frequency is achieved when the rGO conductivity is modified. This finding can be exploited as a detection mechanism for various contemporary applications, including humidity or gas sensing. In order to better illustrate the effect of a change in the rGO properties on the antenna response, a voltage monitor is placed at the antenna source, and the monitored voltage is presented as function of time in Figure 10. Inspecting the outcomes, three different curves are plotted: the blue curve corresponding to the case in which $\sigma_{\mathrm{rGO}}$ changes abruptly within one time-step, along with the the red and yellow ones referring to a linear progressive change of the conductivity within 100 and 500 time-steps, respectively. Despite of the non-instant variation of $\sigma_{\mathrm{rGO}}$, namely when the conductivity is progressively decreased for several time steps, we can still detect a noteworthy oscillation of the voltage, which, essentially, emulates a more realistic scenario than the one with the abrupt conductivity changes, and retains the detection viable for a wide range of material recovery times.

## 4. Conclusions

A new implementation of the PLRC scheme for the accurate modeling of graphene-based materials with time-varying properties has been introduced in this paper. Using this scheme, the rGO has been efficiently modeled and integrated within a patch antenna operating at 6 GHz. Exploiting the transient profile of the FDTD technique, a comprehensive investigation has been performed concerning the influence of rGO conductivity variations on the antenna response. The study, also, enclosed the time modulation of rGO conductivity, which has been utilized to improve the radiation performance of the original antenna. Finally, a sensing mechanism based on the resonance shift induced by graphene’s conductivity variations has been demonstrated.

## Figures and Tables

**Table 1:** Comparison of different FDTD schemes for graphene-based conductive materials.

Implementation	Planar Conductivity	Dispersion (Accuracy)	Time-Varying Properties
Ref. [39]	as impedance	✓ (2nd order)	✗
Ref. [40]	✓	ADE (2nd order)	✗
Ref. [41]	✓	ADE (2nd order)	✗
Ref. [42]	✓	RCM (1st order)	✗
Ref. [43]	✗	PLRC (2nd order)	✗
Ref. [44]	as impedance	✓ (2nd order)	✗
This work	✓	PLRC (2nd order)	✓

(✓): support this property; (✗): does not support this property.

## Data Availability

The data presented in this study are available upon request from the corresponding author (zaharis@auth.gr).
